# The Main Role of Srs2 in DNA Repair Depends on Its Helicase Activity, Rather than on Its Interactions with PCNA or Rad51

**DOI:** 10.1128/mBio.01192-18

**Published:** 2018-07-17

**Authors:** Alex Bronstein, Lihi Gershon, Gilad Grinberg, Elisa Alonso-Perez, Martin Kupiec

**Affiliations:** a School of Molecular Cell Biology and Biotechnology, Tel Aviv University, Ramat Aviv, Israel; University of Texas Health Science Center

**Keywords:** DNA recombination, DNA repair, PCNA, Rad51, Srs2, genome stability, yeasts

## Abstract

Homologous recombination (HR) is a mechanism that repairs a variety of DNA lesions. Under certain circumstances, however, HR can generate intermediates that can interfere with other cellular processes such as DNA transcription or replication. Cells have therefore developed pathways that abolish undesirable HR intermediates. The *Saccharomyces cerevisiae* yeast Srs2 helicase has a major role in one of these pathways. Srs2 also works during DNA replication and interacts with the clamp PCNA. The relative importance of Srs2’s helicase activity, Rad51 removal function, and PCNA interaction in genome stability remains unclear. We created a new *SRS2* allele [*srs2*(*1-850*)] that lacks the whole C terminus, containing the interaction site for Rad51 and PCNA and interactions with many other proteins. Thus, the new allele encodes an Srs2 protein bearing only the activity of the DNA helicase. We find that the interactions of Srs2 with Rad51 and PCNA are dispensable for the main role of Srs2 in the repair of DNA damage in vegetative cells and for proper completion of meiosis. On the other hand, it has been shown that in cells impaired for the DNA damage tolerance (DDT) pathways, Srs2 generates toxic intermediates that lead to DNA damage sensitivity; we show that this negative Srs2 activity requires the C terminus of Srs2. Dissection of the genetic interactions of the *srs2*(*1-850*) allele suggest a role for Srs2’s helicase activity in sister chromatid cohesion. Our results also indicate that Srs2’s function becomes more central in diploid cells.

## IMPORTANCE

Homologous recombination (HR) is a key mechanism that repairs damaged DNA. However, this process has to be tightly regulated; failure to regulate it can lead to genome instability. The Srs2 helicase is considered a regulator of HR; it was shown to be able to evict the recombinase Rad51 from DNA. Cells lacking Srs2 exhibit sensitivity to DNA-damaging agents, and in some cases, they display defects in DNA replication. The relative roles of the helicase and Rad51 removal activities of Srs2 in genome stability remain unclear. To address this question, we created a new Srs2 mutant which has only the DNA helicase domain. Our study shows that only the DNA helicase domain is needed to deal with DNA damage and assist in DNA replication during vegetative growth and in meiosis. Thus, our findings shift the view on the role of Srs2 in the maintenance of genome integrity.

## INTRODUCTION

Homologous recombination (HR) is important for maintaining the stability of the genome; it helps repair double-strand breaks (DSBs) and participates in the recovery of damaged replication forks. However, HR mechanisms can generate intermediates that may block replication forks, or nucleoprotein complexes that can lead to cell cycle arrest and even cause cell death in certain genetic backgrounds (1). That is why HR must be tightly regulated to prevent untimely events that could interfere with other DNA replication or repair mechanisms.

The yeast Saccharomyces cerevisiae is an excellent model to isolate and study mutants that shed light on the processes that maintain genome stability (2, 3). The Srs2 helicase has a major role in HR regulation; it is generally thought that its role is to suppress HR events at an early stage by dismantling the Rad51-presynaptic filament (4, 5). This “antirecombinase” role of Srs2 was first inferred from genetic studies: *srs2* mutants show a hyperrecombination phenotype believed to be caused by an inappropriate channeling of the lesions into the homologous recombination pathway (6–9). The Srs2 protein exhibits single-stranded DNA (ssDNA)-dependent ATPase activity that unwinds DNA with 3′→5′ polarity with a *k*_cat_ of ≥3,000 min^−1^ (10, 11), and the Walker A motif is absolutely required for both ATPase and helicase activities (12). It can unwind a variety of substrates, including those containing forks, flaps, D-loops, 3′ and 5′ single-stranded DNA overhangs, blunt-end double-stranded DNA (dsDNA) substrates, and Holliday junctions (11, 13). Biochemical and electron microscopy analysis revealed that Srs2 can efficiently dismantle the presynaptic filament formed by Rad51, an early HR intermediate (4, 5). It seems that the helicase activity is not responsible for the dissociation, but rather Srs2’s ATP hydrolysis fuels a translocase activity: mutants that cannot bind or hydrolyze ATP fail to disrupt Rad51-presynaptic filaments (12). ATPase mutants show the same sensitivities to genotoxic agents, hyperrecombination phenotype, and genetic interactions as the *srs2* deletion mutant (12). Some studies suggest that Srs2 is guided to the Rad51 filament through a physical interaction with Rad51 (5). Rad51 that cannot interact with Srs2 is resistant to Srs2 antirecombinase activity (14, 15). Other studies suggested that the direct interaction between Srs2 and Rad51 not only targets Srs2 to the HR intermediates but also triggers ATP hydrolysis within the Rad51 filament, causing Rad51 to dissociate from DNA (16). It seems therefore that Srs2 dismantles Rad51 by ATP-driven motor activities of Srs2 that can dissociate both DNA structures and protein-DNA complexes. Recently, it was also shown that Srs2 is able to disrupt extended D-loops created by the activity of polymerase δ (17). Moreover, *in vitro* experiments have shown that Srs2 can unwind structures that resemble D-loops (recombination intermediates) and that this activity is stimulated by Rad51 bound to dsDNA (18).

Srs2 is also needed in the restart of collapsed replication forks together with other members of the Rad6 epistasis group in a process called DNA damage tolerance (DDT). In fact, *SRS2* was first identified because mutations in the gene could suppress the DNA damage sensitivity of both *rad6* and *rad18* mutants (suppressor of RAD six mutant 2), and this suppression requires functional HR (19–24). The main function of the error-free DDT pathway (which includes the Rad6, Rad18, Rad5, Ubc13, and Mms2 proteins) is to ubiquitinate PCNA at its lysine at position 164. If this step is not accomplished, Srs2 is recruited to the replication forks through its binding to SUMOylated PCNA (mediated by adjacent SIM-SUMO-interacting and PIM-PCNA-interacting motifs, which reside at the very end of the protein), and this recruitment seems to prevent HR (23, 25, 26). Thus, when the DDT pathway is impaired, Srs2 activity prevents a possible alternative rescue, resulting in sensitivity to DNA damage. Mutations in *SRS2* seem to open the path for HR and thus suppress the sensitivity of DDT mutants (23, 24).

Although initially Srs2 was considered an inhibitor of HR, later work showed additional roles for Srs2 that favor HR. Srs2 was shown to be required for the repair of DSBs. Cells deleted for *SRS2* show low survival when a single DSB is created, and it appears to act during HR repair, possibly by unwinding the invading strand from the D-loop to allow reannealing with the other broken chromosomal arm (27). Accordingly, Srs2 acts in the promotion of synthesis-dependent strand annealing (SDSA) and inhibition of crossover events (28–30), as well as in additional forms of HR, such as single-strand annealing (SSA), break-induced replication (BIR), as well as in nonhomologous end joining (NHEJ) (31–35).

In recent years, new roles of Srs2 were identified. Srs2 association with SUMOylated PCNA was shown to limit the DNA synthesis by detaching polymerases δ and η from PCNA; this function is independent of the interaction with Rad51 (36). Moreover, Srs2 helicase activity can unwind triplet repeat hairpins at the replication fork; this activity is also independent of Rad51 and plays a critical role in maintaining normal replication without expansion or contraction of repeats (37). Another role for Srs2 is in preventing mutations as a result of Top1 topoisomerase activity on misincorporated ribonucleotides. Srs2 can process the nick after Top1 activity and promote resection by enhancing Exo1 activity. Again, this role of Srs2 was shown to be Rad51 independent (38). Recently, *in vitro* experiments showed that Srs2 can remove replication protein A (RPA) and Rad52-RPA complex from ssDNA, although the *in vivo* significance of these findings is still unclear (39).

Altogether, Srs2 functions as a multifunctional tool that acts in replication, recombination, and DNA repair. By creating a new Srs2 allele that lacks the whole C terminus [*srs2*(*1-850*)], we show here that the DNA helicase domain alone is sufficient to deal with various types of DNA damage, to complete efficient DSB repair, and to promote meiosis. The synthetic lethality (SL) of *Δsrs2* with other deletion mutations is also largely dependent on the DNA helicase activity. The analysis of the SL screen suggests that Srs2 might be involved in sister chromatin cohesion (SCC). Last, we show that the ploidy state of the cell dictates the importance of Srs2’s activity, and diploids rely more on the helicase’s C terminus in order to maintain genome stability.

## RESULTS

### The helicase domain of Srs2 is the major player in dealing with DNA damage.

Our recent study provided evidence that Srs2 has functions that are independent of its role in the eviction of Rad51 and of its interaction with PCNA. Neither the Rad51 interaction region, PIM (PCNA-interacting motif), nor SIM (SUMO-interacting motif), which allow recruitment of Srs2 to SUMOylated PCNA, are required to deal with DNA damage caused by methyl methanesulfonate (MMS) (34).

We were interested in further investigating the importance of the helicase domain for genome stability. To answer this question, we created a new truncation mutation of Srs2 that lacks 324 amino acids (aa) from the C terminus (out of 1,174 aa). This mutation [*srs2*(*1-850*)] lacks all the known interaction sites of Srs2, such as those needed to interact with PCNA, Rad51, Nej1, Mre11, Sgs1, Esc2, Ubc9, Siz1, Siz2, Mus81, Rad5, and Rad18 (23, 40–44). Figure S1 in the supplemental material shows that the *srs2*(*1-850*) strain produces protein, which is expressed from its natural promoter at a slightly higher level than the level produced from the wild type (wt). Importantly, this N-terminal region of Srs2 has been shown to lack the ability to bind Rad51 (15).

10.1128/mBio.01192-18.1 FIG S1  The *srs2*(1-850) allele is expressed, at slightly elevated protein levels. Download FIG S1, TIF file, 0.2 MB. Copyright © 2018 Bronstein et al. 2018 Bronstein et al. This content is distributed under the terms of the Creative Commons Attribution 4.0 International license.

**FIG 1:**
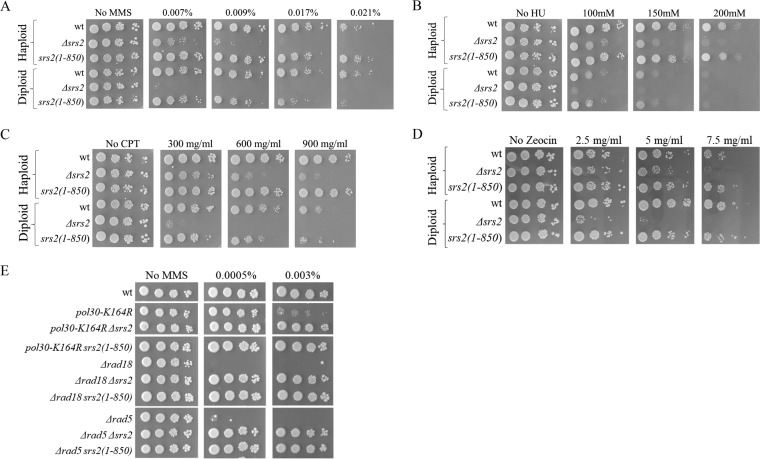
The *srs2*(*1-850*) mutant is fully proficient in dealing with DNA damage. (A to D) The *srs2*(*1-850*) mutant is as resistant as the wt to MMS (A), hydroxyurea (HU) (B), camptothecin (CPT) (C), and zeocin (D). (E) The *srs2*(*1-850*) mutant suppresses the DNA damage sensitivity of impaired DDT mutants.

We first tested how strains carrying this allele handle DNA damage. Figure 1 shows that, surprisingly, the mutants are as proficient as a wt strain when it comes to handling different kinds of DNA damage (MMS [DNA alkylation], hydroxyurea [deoxynucleoside triphosphate {dNTP} depletion], camptothecin [topoisomerase poison], and zeocin [DSBs]). These results imply that Srs2 deals with DNA damage through its helicase region and that the interactions with other proteins are dispensable for its main DNA repair activity. Δ*srs2* mutants are more sensitive to DNA damage as diploid cells than as haploid cells. Consistent with a more central role of HR repair in diploids (34, 45), *Δsrs2* diploids are more sensitive to the DSB-forming agent zeocin. We show that, in contrast, a diploid strain homozygous for the *srs2*(*1-850*) allele is as proficient as the wt parent for growth in the presence of DNA-damaging agents, with the possible exception of MMS, where a barely detected defect can be seen (Fig. 1).

The activity of Srs2 creates toxic intermediates in strains impaired in the DDT pathway and sensitizes them to DNA damage. Deletion of *SRS2* suppresses the DNA damage sensitivity (24). This suppression was shown to be caused by mutations in the C terminus of Srs2 (the SIM and PIM motifs) (23, 42, 46, 47). As expected, the *srs2*(*1-850*) allele (also lacking these motifs) suppressed the MMS sensitivity of *pol30-K164R*, *Δrad18*, and *Δrad5* mutants that are impaired in the DDT pathway (Fig. 1E). Thus, cells with the *srs2*(*1-850*) allele act like the wt when the cells are confronted with external insults to their DNA, but when the DDT pathway is inactivated, it behaves like a mutant with the whole *SRS2* gene deleted. The helicase part of Srs2 is important for dealing with DNA damage; however, when there are no modifications on lysine 164 of PCNA, Srs2 exerts its negative effects through its C terminus, probably via its interactions with PCNA.

### The C terminus of Srs2 is dispensable for Srs2’s role in DNA repair during replication and DSB repair.

**FIG 2:**
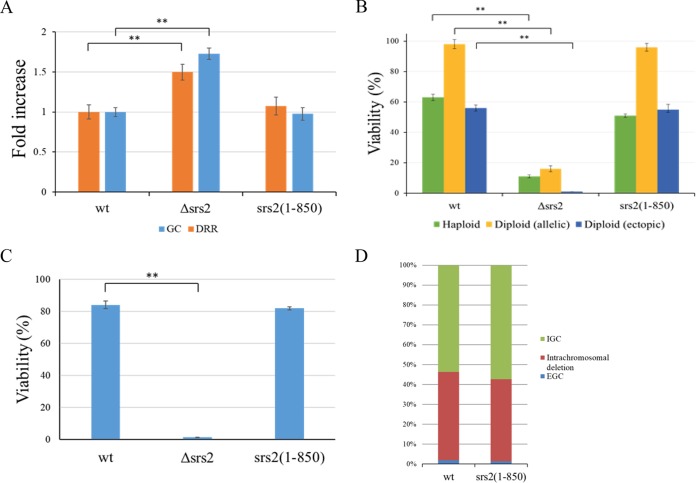
The N terminus of Srs2 is fully proficient in DNA repair of replication damage and DSB repair. (A) Fluctuation tests show that cells with the *srs2*(*1-850*) allele have levels of ectopic gene conversion (GC) and direct-repeat recombination (DRR) similar to those of the wt. (B) A strain that follows the ability to repair a single DSB by allelic or ectopic HR shows that *srs2*(*1-850*), in contrast to Δ*srs2*, acts as a wt in haploids and diploids. (C and D) A strain that measures intrachromosomal recombination and DRR following a single DSB shows that, in contrast to *Δsrs2*, the strain with the allele has the same repair efficiency as the wt. Error bars represent 95% confidence intervals. Asterisks represent *P* values below 0.001. The *P* value between the values for wt and *srs2*(*1-850*) strains was above 0.05 and not statistically different. IGC, interchromosomal gene conversion; EGC, ectopic gene conversion.

Truncation of the C terminus of Srs2 does not sensitize the cells to DNA damage. As Srs2 is involved in several repair pathways, we characterized the repair capacity of the *srs2*(*1-850*) mutant. First, we measured the ability of cells carrying the allele to carry out homologous recombination (48). Strain MK166 allows the measurement of the rates of ectopic gene conversion (GC) and direct-repeat recombination (DRR) during normal cell division (Fig. S2). Relative to the wt, a strain deleted for *SRS2* showed elevated rates of DRR and GC of about 1.5- to 2-fold (49). In contrast, an isogenic strain with the new allele showed levels of both GC and DRR similar to those of the wt (Fig. 2A).

10.1128/mBio.01192-18.2 FIG S2  Schematic representation of strain MK166, in which various forms of homologous recombination can be quantified. Download FIG S2, TIF file, 0.1 MB. Copyright © 2018 Bronstein et al. 2018 Bronstein et al. This content is distributed under the terms of the Creative Commons Attribution 4.0 International license.

Next, we tested whether the *srs2*(*1-850*) mutant is proficient for the repair of a single DSB. Since diploid *srs2*(*1-850*) strains showed some minor sensitivity to MMS, we tested the proficiency of the allele in the repair of an inducible DSB in both haploid and diploid strains.

In the strains used, a single, defined DSB break is created by an inducible HO endonuclease; in the haploid strain, the cells can then repair the damage by an ectopic gene conversion and thus survive and form a colony. Two different diploid strains were used: in the first strain (allelic), the DSB can be repaired by a gene conversion event in which the donor sequence originates at the homologous chromosome. In the second strain (ectopic), the two copies of chromosome V undergo DSBs, and they can be repaired only by recombination with the ectopic donor (Fig. S3). By comparing the number of colonies created when cells are plated on galactose-containing media (continuous HO expression) versus glucose-containing media (no DSB creation), it is possible to calculate the efficiency of repair. Figure 2B shows that wt diploids exhibit an efficiency of repair close to 100% in the presence of an allelic donor. Haploids and diploids that can repair the broken chromosome only by ectopic recombination show about 60% survival. *SRS2* is essential for DSB repair: *Δsrs2* strains exhibit very low repair efficiency in haploid and diploid strains; they are defective for both allelic and ectopic recombination. In contrast, the *srs2*(*1-850*) mutant did not show any significant difference from the wt in any of the three systems tested (Fig. 2B).

10.1128/mBio.01192-18.3 FIG S3  Schematic presentation of the strains used to monitor DSB repair. (A) Haploid strain MK203. Open rectangles represent the *ura3* alleles on chromosomes *II* and *V*. A black box represents the *HOcs*; a gray box depicts the inactive *HOcs-inc* flanked by the BamHI (B) and EcoRI (R) restriction sites. Transfer of the cells to galactose-containing medium results in a DSB that is repaired by ectopic gene conversion. (B) Diploid strain MK235 (allelic) is an isogenic derivative of MK203 in which the DSB (chromosome *V*) can be repaired by recombination with either *URA3* sequences (chromosome *V*) or *ura-Hocs-inc* sequences (chromosome *II*). A gray line depicts the NcoI site in *URA3*. In all our strains, repair took place using the allelic, rather than the ectopic, *ura3* sequences, as donors. (C) The diploid (ectopic) strain is capable of only ectopic gene conversion repair of double-strand breaks in both copies of chromosome *V*. Download FIG S3, TIF file, 0.5 MB. Copyright © 2018 Bronstein et al. 2018 Bronstein et al. This content is distributed under the terms of the Creative Commons Attribution 4.0 International license.

Since *srs2*(*1-850*) does not seem to affect interchromosomal gene conversion, we tested whether it might have an effect on intrachromosomal recombination. Using strain NA3 (50) (Fig. S4), we measured the abilities of the various strains to repair the DSB by intrachromosomal (Rad51 dependent) gene conversion or by single-strand annealing (SSA), which is Rad51 independent. Again, the efficiency of repair is assessed by comparing the number of cells able to form colonies on galactose- versus glucose-based medium. The wt strain showed a repair efficiency of about 85%, whereas the *Δsrs2* mutant had less than 2% repair (Fig. 2C). These results confirm that *SRS2* is also required for intrachromosomal recombination initiated by a DSB. In contrast, the *srs2*(*1-850*) mutant exhibited a repair efficiency similar to that of the wt; furthermore, the distribution between intrachromosomal GC and SSA was similar to that of the wt (Fig. 2D). Taken together, our results show that only the helicase activity of *SRS2* is needed for the repair of DSBs by all types of recombination tested, whereas the C terminus is dispensable.

10.1128/mBio.01192-18.4 FIG S4  Schematic representation of strain NA3. The strain contains an HO endonuclease-cut site within one of intrachromosomal repeats. Below are its repair products following induction in YPGal medium. Download FIG S4, TIF file, 0.5 MB. Copyright © 2018 Bronstein et al. 2018 Bronstein et al. This content is distributed under the terms of the Creative Commons Attribution 4.0 International license.

### The helicase domain of Srs2 is sufficient to undergo proficient meiosis.

The Srs2 protein has a pivotal role in meiotic progression (51). *Δsrs2* diploid cells are unable to undergo a proper meiosis and form few asci. Moreover, most of these asci give rise to dead spores. These defects are caused by the need of Srs2 for efficient homologous recombination in meiosis, which is essential for proper chromosomal segregation during the meiotic divisions (51).

**FIG 3:**
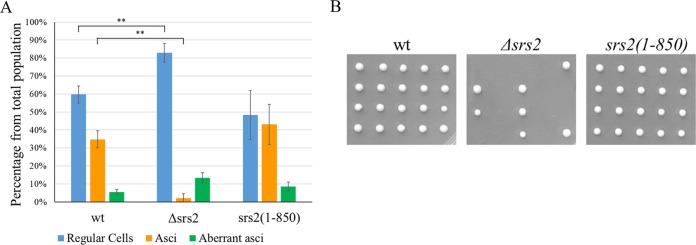
Function of the *srs2*(*1-850*) allele in meiosis. (A) *srs2*(*1-850*) strain is fully capable to undergo meiosis. (B) *srs2*(*1-850*) strain is fully capable to form viable colonies. Error bars represent the 95% confidence intervals. Asterisks represent *P* values below 0.001. The *P* value between wt and *srs2*(*1-850*) was above 0.05 and not statistically different.

To test the *srs2*(*1-850*) allele for possible meiotic defects, we subjected wt, *Δsrs2*/*Δsrs2*, and *srs2*(*1-850*)/*srs2*(*1-850*) diploids to meiosis. Diploid cells were allowed to sporulate for 6 days, and the percentage of cells that completed meiosis to form asci was determined. *Δsrs2* homozygotes formed very few asci, and most of the spores from these asci were unable to form colonies (Fig. 3A). Homozygous *srs2*(*1-850*) diploids, in contrast, showed no impairment in meiosis. Tetrad dissection showed that, contrary to what is seen in *Δsrs2*/*Δsrs2* strains, viability of the *srs2*(*1-850*)/*srs2*(*1-850*) spores was as high as that of the wt spores (Fig. 3B). We conclude that *srs2*(*1-850*) does not have meiotic defects, again pointing to Srs2’s helicase domain as the main need for Srs2’s activity during meiosis.

### Genetic interactions of the *Δsrs2* and *srs2*(*1-850*) alleles.

The data we showed so far provide strong evidence for the hypothesis that the main role of Srs2 in genome stability is carried out by the helicase activity present at its N terminus and does not require interactions with additional repair proteins or with PCNA. Systematic screens (52–54) have shown that there is a large number of genes that, when deleted, show a dependence on Srs2 function for survival. For most of the genes, it is unknown why, if mutated, they are synthetic sick or lethal with *Δsrs2*. We saw an opportunity to identify the regions of Srs2 that are responsible for the impaired growth in these genetic backgrounds. We chose genes involved in DNA metabolism that, when deleted, show the most severe negative interactions with the *Δsrs2* allele. Diploid strains heterozygous for various deletion allele and for *srs2*(*1-850*) or *Δsrs2* were subjected to meiosis, and tetrads were dissected. The viability and growth rate of double mutant spores defective for each of the chosen genes and carrying the *srs2*(*1-850*) allele was compared to that of the double mutant with *Δsrs2*. The results (Table 1) are grouped according to their phenotypes. We could distinguish three categories.

**TABLE 1:**
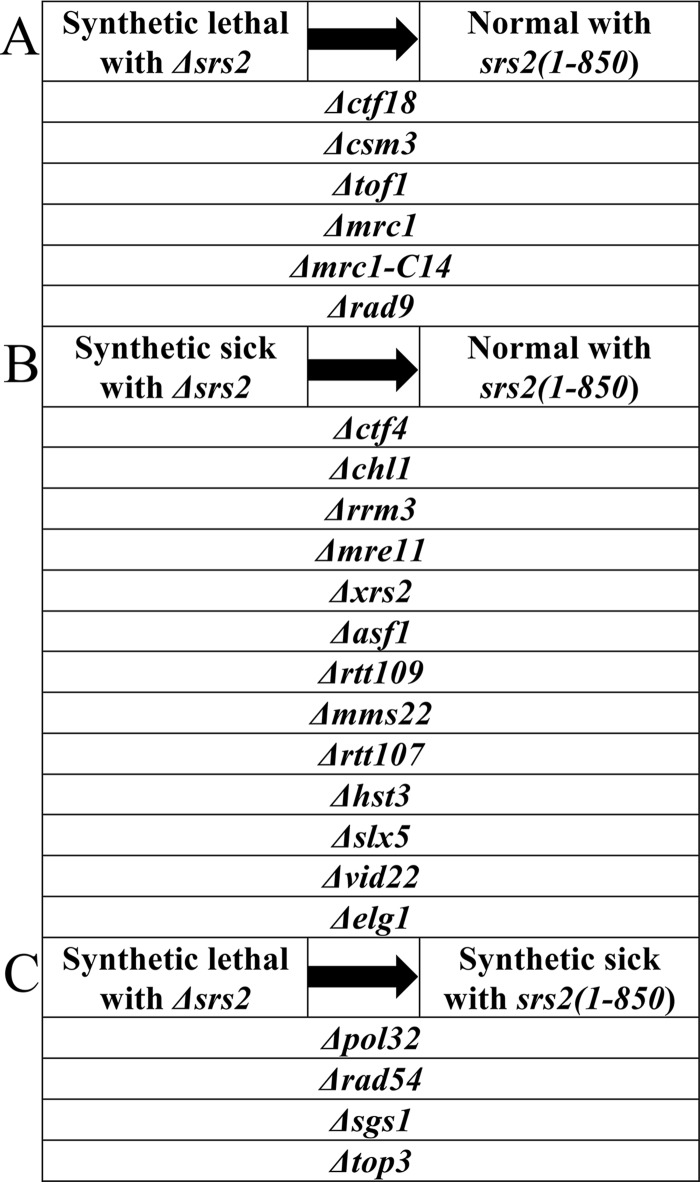
Summary of synthetic interactions with *Δsrs2* and *srs2*(*1-850*)^a^

a A synthetic sick phenotype is observed when double mutant spores generate colonies smaller than single mutants; synthetic lethality is when spores are unable to generate colonies at all. At least 14 tetrads where dissected for each mutation combination.

**FIG 4:**
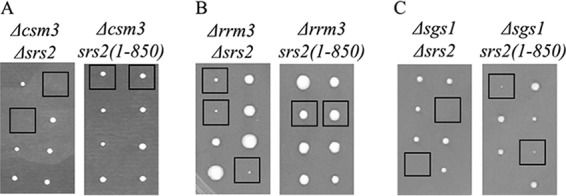
Genetic analysis of genes which are synthetic sick with *Δsrs2* but grow normally with *srs2*(*1-850*). (A to C) Examples of different phenotypes observed when crossed with the *SRS2* allele.

1. The first set of deleted genes showed synthetic lethality with *Δsrs2* but grew normally when combined with *srs2*(*1-850*) (Table 1A and Fig. 4A).

- **Ctf18.** Ctf18 is part of a replication factor C (RFC)-like complex that moves with the replication fork and participates in sister chromatid cohesion and checkpoint response (55).
- **Csm3.** Csm3, together with Tof1, is located at the replication fork, where it contributes to fork stability by inhibiting fork rotation caused by topological stress of unwinding the dsDNA ahead of the polymerases (56).
- **Mrc1.** Mrc1 is involved in replication checkpoint activation; it mediates phosphorylation of Rad53 by Mec1 and is active through S phase. It is also required for proper DNA replication (57, 58). The synthetic lethality between *Δmrc1* and *Δsrs2* was found to be independent of the checkpoint activity of *MRC1*: the *mrc1-AQ* allele, defective in the checkpoint function of *MRC1*, does not show synthetic sickness or lethality with *Δsrs2* (53). Site-specific mutations of *MRC1* were found to impair specifically its role at the replication fork but not in DNA damage signaling (59). We crossed one such *MRC1* mutant (*mrc1-C14*) with *Δsrs2* and observed synthetic lethality (SL). However, when combined with *mrc-C14*, the *srs2*(*1-850*) allele led to normal cell growth (Table 1A). We thus conclude that the DNA replication functions of Mrc1 and the helicase activity of Srs2 are synthetic lethal, and not the other functions of these proteins.
- **Rad9.** Rad9 is a checkpoint adapter, which is required throughout the cell cycle (60–62).

2. A second set of genes showed synthetic sickness (but not lethality) with *Δsrs2* and normal growth with *srs2*(*1-850*) (Table 1B and Fig. 4B).

- **Ctf4 and Chl1.** The Ctf4 and Chl1 proteins are involved in sister chromatid cohesion and genome integrity and interact with Ctf18 (63, 64).
- **Rrm3.** Rrm3 is another helicase that assists in the replication of regions of the genome with secondary structures or bound proteins (65, 66).
- **Mre11 and Xrs2.** The Mre11 nuclease and Xrs2, together with Rad50, form the MRX complex, which is important for end resection after DSB creation and for replication fork stability (67).
- **Histone acetylation proteins Asf1, Rtt109, Mms22, and Rtt107.** *ASF1*, *RTT109*, *MMS22* and *RTT107* encode proteins that are involved in the acetylation of newly deposited histones (marked by an acetyl group in the lysine 56 of histone H3 [H3K56]) (54). This acetylation is important for proper DNA replication and DNA damage response (68).
- **Hst3.** Hst3 is the histone deacetylase that removes the acetyl groups from H3K56 (69). Surprisingly, both lack of acetylation and too much acetylation have a similar synthetic sick phenotype in the absence of Srs2 helicase activity.
- **Slx5.** Slx5 forms with Slx8 a SUMO-targeted ubiquitin (Ub) ligase (STUbL) complex that attaches ubiquitin to poly-SUMOylated proteins, supposedly in order to send them for degradation during replication and DNA repair (70).
- **Vid22.** Vid22 acts as a chromatin remodeler and removes nucleosome from DNA damage repair site. This in turn allows the recruitment of the MRX complex, which initiate the repair (71).
- **Elg1.** The RFC-like complex composed of Elg1 and four of the small subunits of RFC unloads PCNA during DNA replication and repair (72–74).

3. A third group of mutants were synthetic lethal with *Δsrs2* and showed synthetic sickness with the *srs2*(*1-850*) allele (Table 1C and Fig. 4C). This implies that both Srs2’s helicase activity and the C terminus of Srs2 are required for proper cell activity in these genetic backgrounds.

- **Pol32.** Pol32 is a subunit of polymerase δ required for efficient DNA synthesis and BIR (break-induced replication) repair (75, 76).
- **Rad54.** Rad54 is a chromatin remodeling factor that is needed for DSB repair; it participates in D-loop formation, extension, and resolution (77).
- **Sgs1, Rmi1, and Top3.** The Sgs1, Rmi1, and Top3 proteins form a complex required for many different aspects of genome stability and DNA repair, including DNA resection, the resolution of Holiday junction intermediates, and the relaxation of supercoiled DNA (78).

Taken together, the results point to the fact that in the absence of Srs2 function, histone deposition, checkpoint activation, and sister chromatid cohesion become impaired (see Discussion).

### Ploidy dictates Srs2 activity.

Previous results have shown that in certain genetic backgrounds (for example, in the absence of the Elg1 RFC-like subunit), deletion of *SRS2* has little effect in haploids, but diploids fail to form colonies (72). We therefore tested the double mutants that showed normal growth as haploids for their phenotype as diploids. Double mutants were mated, and the homozygous diploid zygotes (>24 per strain) were manipulated to predetermined locations on rich medium plates. All mutants that showed synthetic sickness with *Δsrs2* were not viable as diploids and generated no, or only a few, viable colonies. Thus, all the genetic interactions of *Δsrs2* are stronger in diploids than in haploids. In contrast, most of the combinations of the *srs2*(*1-850*) allele and various deletions that showed normal growth as haploids were able to form viable diploids (although in some cases only 2/3 of the zygotes grew). The striking exceptions to this rule were diploids homozygous for the *srs2*(*1-850*) allele and for *Δrad54*, *Δpol32*, Δ*sgs1*, or *Δtop3*. Whereas these double mutant strains grew slowly as haploids, they failed to form diploid colonies, similarly to the double mutants with *Δsrs2*. This illustrates that in certain situations, the C terminus of Srs2 becomes important in diploids (see Discussion).

In conclusion, Srs2 has various roles in DNA replication and chromosome maintenance, which depend on the genome state of the cell. In haploids and diploids, the DNA helicase activity of *SRS2* is required for supporting proper DNA replication and chromosome segregation. However, in some genetic backgrounds (such as in the absence of Pol32, Rad54, or Sgs1), the C terminus is also important for reliable DNA replication in diploids.

## DISCUSSION

The *srs2* mutant was originally isolated as a suppressor of the DNA damage sensitivity of mutants with an impaired DDT pathway; genetic evidence suggested that this suppression depends on Rad51 (6, 8, 24). *In vitro* experiments showed that Srs2 is able to disrupt Rad51 nucleofilaments (4, 5) and inhibit recombination at D-loops and replication forks by binding to PCNA (4, 5, 23). After these convincing biochemical experiments, it was widely assumed that the role of Srs2 in the maintenance of genomic integrity is to inhibit recombination by removing Rad51 from the DNA. Thus, all phenotypes of *Δsrs2* were interpreted in light of this activity.

Although many times described as an “antirecombinase,” Srs2 is essential for DSB repair by HR (27); thus, its activity is both pro- and antirecombinational (44). Moreover, even Srs2 alleles that lack the region required for interactions with Rad51 or PCNA (or with any of the proven Srs2 interactors) are still proficient in promoting synthesis-dependent strand annealing (SDSA) over crossover resolution (79) and perfectly complement the sensitivity of *Δsrs2* mutants to DNA damage (34, 37, 38, 80 this work). The new *srs2* allele [*srs2*(*1-850*)], which lacks the entire C terminus and has only the DNA helicase domain (15), is unable to interact with Rad51 (15) or with any of the known partners of Srs2 (PCNA, Rad51, Nej1, Mre11, Sgs1, Esc2, Ubc9, Siz1, Siz2, Mus81, Rad5, and Rad18 [23, 40–44]). We showed that *in vivo*, the helicase of Srs2 was enough to fully deal with various DNA-damaging agents and with HO-induced DSB in both haploids and diploids. Mitotic recombination, meiosis progression, and spore survival were also unaffected. Only when the DDT pathway was impaired was the helicase domain insufficient to enable viability. Thus, binding to PCNA through the C-terminal PIM and SIM motifs becomes essential in the absence of the PCNA ubiquitination that allows DDT pathways to work. Our work thus defines two separate domains of the Srs2 protein with different biological relevance.

### Srs2 supports sister chromatid cohesion.

Srs2 plays an important role during DNA replication and chromosome segregation, as evidenced by strong negative genetic interactions with mutants defective in these processes. Our analysis showed that the *srs2*(*1-850*) allele is less affected than the *Δsrs2* allele to inactivation of additional DNA processing functions. This implies that the helicase activity of Srs2 is sufficient for normal growth in most of the mutant backgrounds.

Csm3, Tof1, Ctf18, Ctf4, Mrc1, Slx5, and Elg1 have many diverse roles in keeping genome stability. Analyzing the common role between these proteins revealed that they all have a function in sister chromatid cohesion (SCC) (53, 55, 64, 81–84). In addition to the DNA helicases (Chl1, Sgs1, and Rrm3), Mre11 and Srs2 are also involved in SCC (85, 86).

Interestingly, *Δsrs2* and *Δmre11* synthetic lethality was not dependent on the nuclease activity of the MRX complex (which is necessary for MRX’s role in end resection) or on active HR (87, 88). These results suggest that Srs2 and Mre11 are required for proper DNA replication, but not in their classical role of repairing the DNA damage during replication. Thus, their alternative role in DNA replication could also be in SCC, as the MRX complex has been shown to affect this process (89).

Asf1, Rtt109, Rtt107, Hst3, and Mms22 also have a role in SCC, as histone acetylation metabolism was found to act in the regulation of SCC (90–92). It seems that proper regulation of histone H3 acetylation is important for chromosome cohesion and segregation.

Two nonessential pathways were proposed to promote SCC. The first pathway is composed of Tof1, Csm3, Ctf4, and Chl1, and the second pathway is composed of Mrc1, the Ctf18 RFC-like complex, and the Sgs1-Top3-Rmi1 complex (82, 93). Srs2 does not seem to belong specifically to one of the nonessential SCC pathways. Rather, it has a supporting role for the two SCC pathways. This is evident also by the supporting role of Srs2 in histone acetylation metabolism during SCC. The role of helicases in SCC is unknown; it was suggested that helicases might prepare the DNA for targeting of new cohesin rings by removing old cohesin units left on the DNA and by stimulating the loading of new cohesins during replication (94, 95). These functions could be executed by Srs2’s DNA helicase and translocase capabilities.

### The C terminus of Srs2 is required for specific functions during DNA replication to promote genome integrity.

In contrast to the previous lack of synthetic phenotypes, the *srs2*(*1-850*) allele was synthetic sick when combined with *Δsgs1*, *Δpol32*, or *Δrad54*.

Both Pol32 and Srs2 are implicated in BIR (76). BIR is divided into two pathways: a Rad51-dependent branch and a Rad51-independent branch (96). In the absence of *POL32*, the C terminus of Srs2 is partly required for cell viability. The C terminus contains the Rad51-interacting motif, a fact that may implicate Srs2 in the Rad51-dependent BIR. Alternatively, it may be the interaction of Srs2 to PCNA that is required. When the Srs2 pathway is disrupted, cells become completely dependent on the Pol32-mediated repair pathway (97).

The synthetic lethality between *Δsrs2* and *Δrad54* is more complex. It has been proposed that Srs2 and Rad54 actually act in the same pathway, and the SL interaction is due to the generation of toxic intermediates that are trapped and making the cells unable to proceed with the repair without Rad54, but the generated intermediates also cannot recede to an alternative repair pathway due to lack of Srs2 antirecombinase activity (98). This is consistent with our results showing that the C terminus (Rad51 interaction region) of Srs2 is required for proper DNA replication in *Δsrs2 Δrad54* cells. A similar explanation could also be applied to the synthetic sickness of the *srs2*(*1-850*) allele in the absence of a functional Sgs1 helicase. Sgs1’s activity affects many stages of the HR process, from resection to resolution, as well as having a role in SCC (99).

### Ploidy regulates a wider range of Srs2’s activities.

All of the double mutants with *Δsrs2* that are synthetic sick as haploid cells become essential in diploid cells. The DNA helicase of Srs2 is, however, sufficient to suppress the SL phenotype in diploids. This implies that the DNA helicase part of Srs2 becomes more central in diploids and is crucial for cell viability in the absence of other factors (72). In certain genetic backgrounds, the C terminus of Srs2 also becomes important in diploids. Mutants that are synthetic sick with *srs2*(*1-850*) as haploids (*Δrad54*, *Δpol32*, and *Δsgs1*) are inviable as diploids. Ploidy seems to affect the fundamental regulation of the pathways involved in dealing with DNA replication stress. Haploids rely more on the DDT pathways, whereas diploids seem to rely more on HR (34, 45). It seems that PCNA and its modifications affect the regulation of DNA repair during replication, depending on the ploidy of the cell. The fact that diploids rely more on HR to deal with DNA damage is consistent with our finding that Srs2 C terminus and probably its antirecombinase activity is important in diploids, when other factors of HR are unavailable.

In conclusion, we show that the helicase activity of Srs2, and not its physical interactions with Rad51 or PCNA, plays a major role in genome maintenance. PCNA interaction becomes important only in the absence of the DDT pathway. We also show that Srs2 plays a role in SCC and that its helicase activity becomes more important in diploid cells.

## MATERIALS AND METHODS

### Yeast strains.

*Saccharomyces cerevisiae* strains used in this study are listed in Table 2. Unless otherwise stated, strains used were of one of these backgrounds.

- **MK166:** *MAT**a** lys2*::*Ty1Sup ade2-1*(*o*) *can1-100*(*o*) *ura3-52 leu2-3*,*112 his3del200 trp1del901 HIS3*::*lys2*::*ura3 his4*::*TRP1*::*his4* (48).
- **MK203:** *MAT**a**-inc ura3*::*HOcs* (*V*) *lys2*::*ura3-HOcs inc* (1.2 kb) *ade3*::*GALHO leu2-3*,*112 his3-11*,*15 trp1-1 ade2-1 can1-100* (100). This strain is based on W303 (27).
- **NA3:** MK203 carrying pM53 (*URA3*^*+*^ [1.2 kb] *TRP1*^*+*^ [1.4 kb]) integrated into *ura3*::*HOcs* and an additional donor in *lys2.* The genotype of strain NA3 is *MAT**a**-inc ade2 ade3*::*GALHO ura3HOcs ---TRP1 ---URA3* (1.2 kb) *leu2-3*,*112 his3-11*,*13 trp1-1 lys2*::*ura3*::*HOcs-inc* (50).
- **Sch2:** *MAT**a**/MAT*α *ura3*::*HOcs/URA3* (*V*) *lys2*::*ura3*::*HOcs-incRB* (1.2 kb)*/LYS2* (*II*)*ade3*::*GALHO leu2-3*,*112 his3-11*,*15 trp1-1 ade2-1*. (This strain is based on strain W303.)
- **Sch4:** *MATa/MAT*α *ura3*::*HOcs* (*V*) *lys2*::*ura3*::*HOcs-incRB* (1.2 kb) *ade3*::*GALHO leu2-3*,*112 his3-11*,*15 trp1-1 ade2-1 can1-100.* (This strain is based on strain W303.)

**TABLE 2:** Yeast strains used in this study

Strain	Relevant genotype	Reference or source
MK166 diploid	*MAT**a**/MAT*α	48
AB101	MK166 *MAT**a***	48
AB217	MK166 *MAT**a** mrc1*::*natR*	This study
AB91	MK166 *MAT**a** rad9*::*natR*	This study
op883	MK166 *MAT**a** srs2*::*KanMX*	49
AB270	MK166 *MAT**a** pol30-K164R*::*KanMX srs2*::*KanMX*	This study
op710	MK166 *MAT*α *elg1*::*HygMX*	Lab stock
op952	MK166 *MAT**a** pol30-K164R*::*LEU2*	Lab stock
AB106	MK166 *MAT*α *pol30-K164R*::*LEU2 srs2*::*KanMX*	This study
AB365	MK166 diploid *srs2*::*KanMX*	This study
AB366	MK166 diploid *srs2*(*1-850*)::*HygMX*	This study
AB298	MK166 *MAT**a** srs2*(*1-850*)::*HygMX*	This study
OP1122	MK166 *MAT**a** rad18*::*LEU2*	49
op890	MK166 *MAT**a** rad5*::*KanMX*	49
OP1125	MK166 *MAT**a** rad18*::*LEU2 srs2*::*KanMX*	101
AB234	MK166 *MAT**a** rad5*::*KanMX srs2*::*KanMX*	101
AB353	MK166 *MAT**a** srs2*(*1-850*)::*HygMX rad18*::*LEU2*	This study
AB339	MK166 *MAT**a** srs2*(*1-850*)::*HygMX pol30-K164R*::*KanMX*	This study
AB341	MK166 *MAT**a** srs2*(*1-850*)::*HygMXrad5*::*KanMX*	This study
MK203	*MAT**a***	Lab stock
MK15514	MK203 *srs2*(*1-850*)::*HygMX*	This study
SIJB16	MK203 *srs2*::*KanMX*	Lab stock
NA3	*MAT**a***	Lab stock
SIJB30	NA3 *srs2*::*LEU2*	This study
MK15519	NA3 *srs2*(*1-850*)::*HygMX*	This study
Sch2	*MAT**a**/MAT*α	Lab stock
Sch4	*MAT**a**/MAT*α	Lab stock
MK11208B	*Sch2 srs2*::*LEU2*	Lab stock
MK15575	Sch2 *srs2*(*1-850*)::*HygMX*	This study
MK13120	Sch4 *srs2*::*LEU2*	Lab stock
MK15576	Sch4 *srs2*(*1-850*)::*HygMX*	This study
MK17285	MK166 *MAT**a** csm3*::*KanMX*	This study
MK17297	MK166 *MAT**a** csm3*::*KanMX srs2*(*1-850*)::*HygMX*	This study
MK17298	MK166 *MAT*α *csm3*::*KanMX srs2*(*1-850*)::*HygMX*	This study
MK17323	MK166 *MAT**a** mrc1*::*natR srs2*(*1-850*)::*HygMX*	This study
MK17325	MK166 *MAT*α *mrc1*::*natR srs2*(*1-850*)::*HygMX*	This study
AB297	MK166 *MAT**a** srs2*(*1-850*)::*HygMX*	This study
AB331	MK166 *MAT*α *srs2*(*1-850*)::*HygMX*	This study
MK4252	MK166 *MAT**a** ctf18*:::*HygMX*	Lab stock
AB386	MK166 *MAT*α *ctf18*::Hyg *srs2*::*KanMX*	This study
AB388	MK166 *MAT**a** ctf18*::*HygMX srs2*::*KanMX*	This study
AB390	MK166 *MAT*α *ctf18*::*HygMX srs2*(*1-850*)::*HygMX*	This study
AB392	MK166 *MAT**a** ctf18*::*HygMX srs2*(*1-850*)::*HygMX*	This study
AB367	MK166 *MAT**a** srs2*::*KanMX elg1*::*HygMX*	This study
AB368	MK166 *MAT**a** srs2*::*KanMX elg1*::*HygMX*	This study
AB369	MK166 *MAT**a** srs2*(*1-850*)::*HygMX elg1*::*HygMX*	This study
AB3670	MK166 *MAT*α *srs2*(*1-850*)::*HygMX elg1*::*HygMX*	This study
MK7232	MK166 *MAT**a** rrm3*::*KanMX*	Lab stock
AB379	MK166 *MAT*α *rrm3*::*KanMX srs2*::*HygMX*	This study
AB381	MK166 *MAT**a** rrm3*::*KanMX srs2*::*HygMX*	This study
AB382	MK166 *MAT*α *rrm3*::*KanMX srs2*(*1-850*)::*HygMX*	This study
AB384	MK166 *MAT**a** rrm3*::*KanMX srs2*(*1-850*)::*HygMX*	This study
op1149	MK166 *MAT**a** ctf4*::*KanMX*	Lab stock
AB394	MK166 *MAT*α *ctf4*::*KanMX srs2*::*HygMX*	This study
AB396	MK166 *MAT**a** ctf4*::*KanMX srs2*::*HygMX*	This study
AB398	MK166 *MAT**a** ctf4: KanMX srs2*(*1-850*)::*HygMX*	This study
AB400	MK166 *MAT*α *ctf4*::*KanMX srs2*(*1-850*)::*HygMX*	This study
AB417	MK166 *MAT**a** rtt109*::*KanMX*	This study
AB424	MK166 *MAT**a** rtt109*::*KanMX srs2*::*HygMX*	This study
AB426	MK166 *MAT*α *rtt109*::*KanMX srs2*::*HygMX*	This study
AB428	MK166 *MAT**a** rtt109*::*KanMX srs2*(*1-850*)::*HygMX*	This study
AB430	MK166 *MAT*α *rtt109*::*KanMX srs2*(*1-850*)::*HygMX*	This study
AB421	MK166 *MAT**a** xrs2*::*KanMX*	This study
AB457	MK166 *MAT**a** xrs2*::*KanMX srs2*(*1-850*)::*HygMX*	This study
AB459	MK166 *MAT*α *xrs2*::*KanMX srs2*(*1-850*)::*HygMX*	This study
AB461	MK166 *MAT**a** xrs2*::*KanMX srs2*::*HygMX*	This study
AB463	MK166 *MAT*α *xrs2*::*KanMX srs2*::*HygMX*	This study
MK4097	MK166 *MAT**a** mre11*::*KanMX*	This study
AB432	MK166 *MAT**a** mre11*::*KanMX srs2*::*HygMX*	This study
AB434	MK166 *MAT*α *mre11*::*KanMX srs2*::*HygMX*	This study
AB436	MK166 *MAT**a** mre11*::*KanMX srs2*(*1-850*)::*HygMX*	This study
AB438	MK166 *MAT*α *mre11*::*KanMX srs2*(*1-850*)::*HygMX*	This study
MK12598	MK166 *MAT*α *chl1*::*KanMX*	Lab stock
AB440	MK166 *MAT**a** chl1*::*KanMX srs2*(*1-850*)::*HygMX*	This study
AB442	MK166 *MAT*α *chl1*::*KanMX srs2*(*1-850*)::*HygMX*	This study
MK7267	MK166 *MAT**a** asf1*::*KanMX*	Lab stock
AB444	MK166 *MAT**a** asf1*::*KanMX srs2*::*HygMX*	This study
AB446	MK166 *MAT*α *asf1*::*KanMX srs2*::*HygMX*	This study
AB448	MK166 *MAT**a** asf1*::*KanMX srs2*(*1-850*)::*HygMX*	This study
AB450	MK166 *MAT*α *asf1*::*KanMX srs2*(*1-850*)::*HygMX*	This study
AB371	MK166 *MAT**a** srs2*::*HygMX*	This study
AB372	MK166 *MAT*α *srs2*::*HygMX*	This study
MK7781	MK203 *MAT**a** vid22*::*NatR*	Lab stock
AB475	MK203 *MAT**a** vid22*::*NatR srs2*::*LEU2*	This study
AB477	MK203 *MAT*α *vid22*::*NatR srs2*::*LEU2*	This study
AB479	MK203 *MAT**a** vid22*::*NatR srs2*(*1-850*)::*HygMX*	This study
AB481	MK203 *MAT*α *vid22*::*NatR srs2*(*1-850*)::*HygMX*	This study
AB423	MK166 *MAT**a** slx5*::*KanMX*	This study
AB503	MK166 *MAT**a** slx5*::*KanMX srs2*::*HygMX*	This study
AB505	MK166 *MAT*α *slx5: KanMX srs2*::*HygMX*	This study
AB507	MK166 *MAT**a** slx5*::*KanMX srs2*(*1-850*)::*HygMX*	This study
AB509	MK166 *MAT*α *slx5*::*KanMX srs2*(*1-850*)::*HygMX*	This study
MK14408	MK166 *MAT**a** pol32*::*KanMX*	Lab stock
AB411	MK166 *MAT*α *srs2*(*1-850*)::*HygMX pol32*::*KanMX*	This study
AB413	MK166 *MAT**a** srs2*(*1-850*)::*HygMX pol32*::*KanMX*	This study
AB134	MK166 *MAT**a** rad54*::*KanMX*	This study
AB401	MK166 *MAT*α *rad54*::*KanMX srs2*(*1-850*)::*HygMX*	This study
AB403	MK166 *MAT**a** rad54*::*KanMX srs2*(*1-850*)::*HygMX*	This study
AB405	MK166 *MAT*α *sgs1*::*KanMX srs2*(*1-850*)::*HygMX*	This study
AB407	MK166 *MAT**a** sgs1*::*KanMX srs2*(*1-850*)::*HygMX*	This study
MK4137	MK166 *MAT**a** sgs1*::*KanMX*	Lab stock
17371	MK166 *MAT**a** mrc1-C14*::*KanMX*	This study
17376	MK166 *MAT**a** mrc1-C14*::*KanMX srs2*(*1-850*)::*HygMX*	This study
17377	MK166 *MAT*α *mrc1-C14*::*KanMX srs2*(*1-850*)::*HygMX*	This study
AB491	MK166 *MAT**a** top3*::*LEU2*	This study
17396	MK166 *MAT**a** hst3*::*KanMX*	This study
17420	MK166 *MAT*α *hst3*::*KanMX srs2*::*HygMX*	This study
17421	MK166 *MAT**a** hst3*::*KanMX srs2*::*HygMX*	This study
17424	MK166 *MAT*α *hst3*::*KanMX srs2*(*1-850*)::*HygMX*	This study
17425	MK166 *MAT**a** hst3*::*KanMX srs2*(*1-850*)::*HygMX*	This study
17428	MK166 *MAT**a** rad9*::*KanMX srs2*::*HygMX*	This study
17432	MK166 *MAT*α *rad9*::*KanMX srs2*::*HygMX*	This study
17432	MK166 *MAT**a** rad9*::*KanMX srs2*(*1-850*)::*HygMX*	This study
17433	MK166 *MAT*α *rad9*::*KanMX srs2*(*1-850*)::*HygMX*	This study
17388	MK166 *MAT**a** rtt107*::*KanMX*	This study
17436	MK166 *MAT**a** rtt107*::*KanMX srs2*::*HygMX*	This study
17437	MK166 *MAT*α *rtt107*::*KanMX srs2*::*HygMX*	This study
17440	MK166 *MAT**a** rtt107*::*KanMX srs2*(*1-850*)::*HygMX*	This study
17441	MK166 *MAT*α *rtt107*::*KanMX srs2*(*1-850*)::*HygMX*	This study

Standard yeast molecular genetic techniques were used to delete individual genes.

### Determination of recombination rates.

Strain MK166 carries substrates that allow easy scoring of direct-repeat recombination (DRR) (His^+^ colonies) and ectopic gene conversion (GC) (Lys^+^ colonies). Colonies isolated from plates with various concentrations of methyl methanesulfonate (MMS) were subjected to fluctuation tests, and the rates were calculated as described previously (48). The MMS concentrations used were low and did not cause cell death in the wild-type (wt) strain.

### Repair efficiency measurement.

NA3, MK203, Sch2, and Sch4 strain derivatives were streaked onto yeast extract-peptone-dextrose (YPD) plates. Individual colonies were resuspended in water, appropriately diluted, and plated on YPD and yeast extract-peptone-galactose (YPGal) plates. The colonies were counted after 3 days of incubation at 30°C (27, 50).
