# Supplementary figures and images for: The Main Role of Srs2 in DNA Repair Depends on Its Helicase Activity, Rather than on Its Interactions with PCNA or Rad51
Source: mBio. 2018 Jul 17;9(4):e01192-18. doi: 10.1128/mBio.01192-18 (PMC6050964; doi:10.1128/mBio.01192-18)

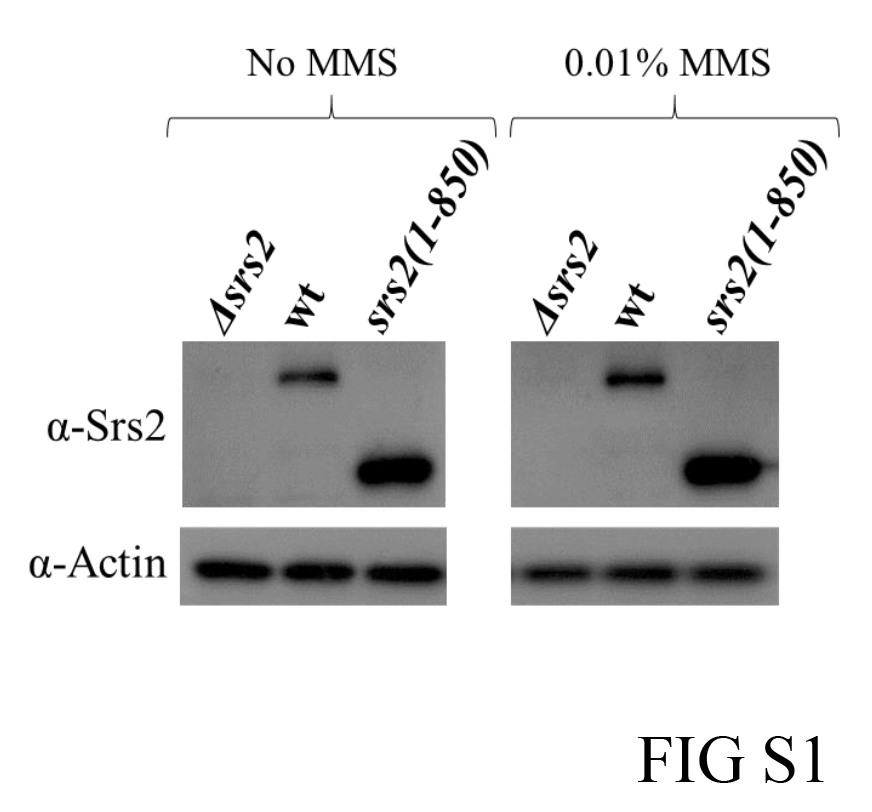

Supplement: FIG S1 [file mbo004183980sf1.tif]

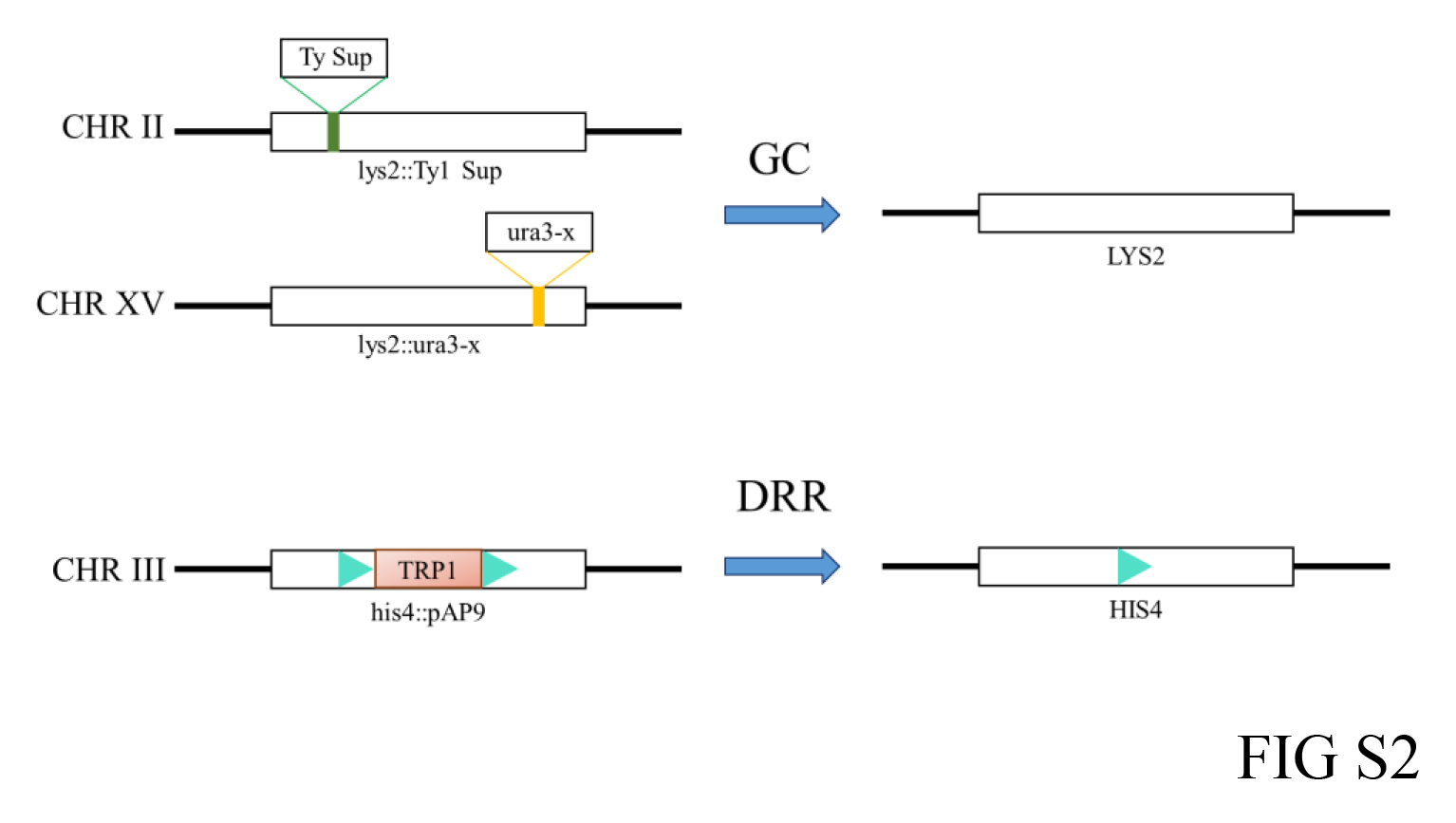

Supplement: FIG S2 [file mbo004183980sf2.tif]

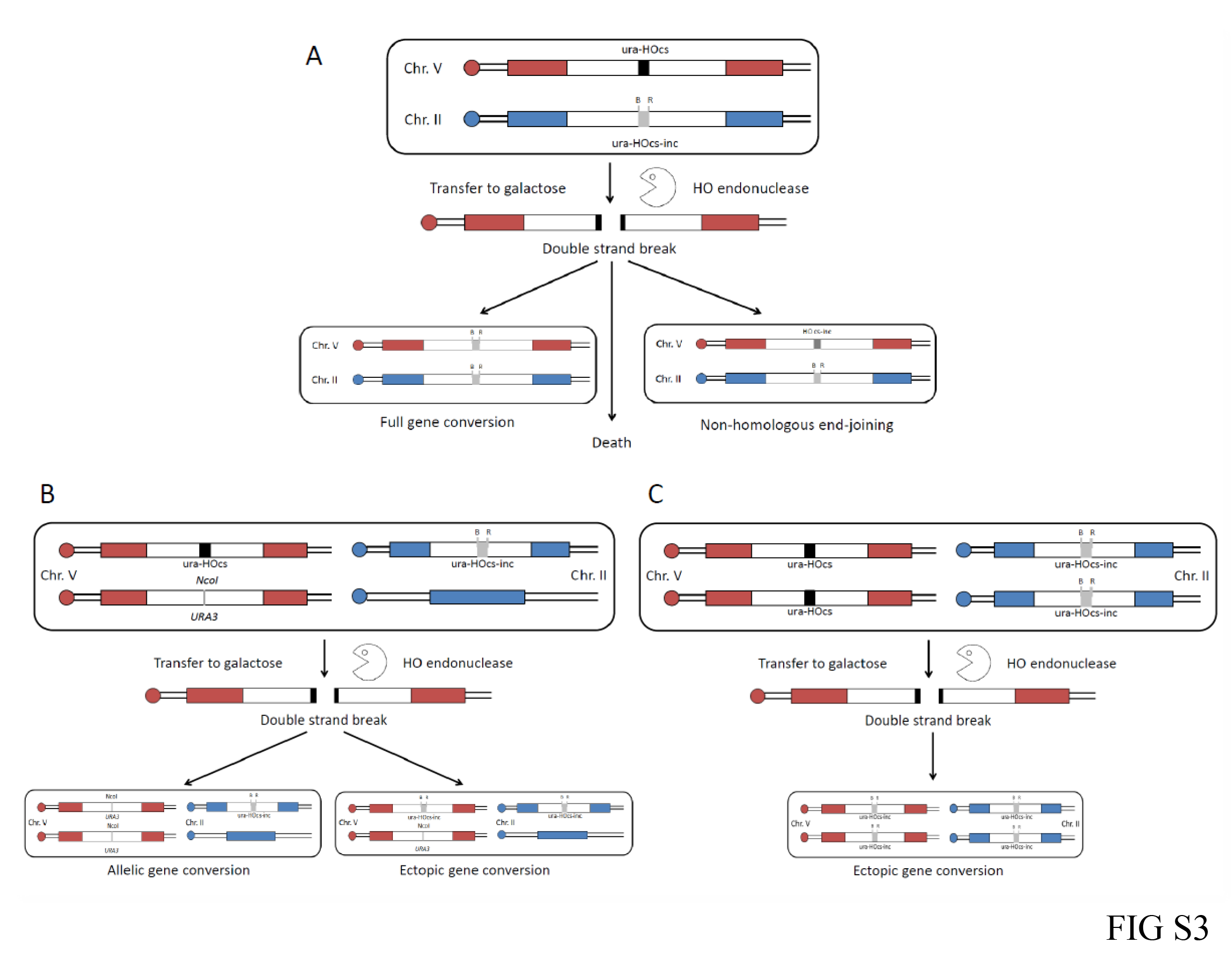

Supplement: FIG S3 [file mbo004183980sf3.tif]

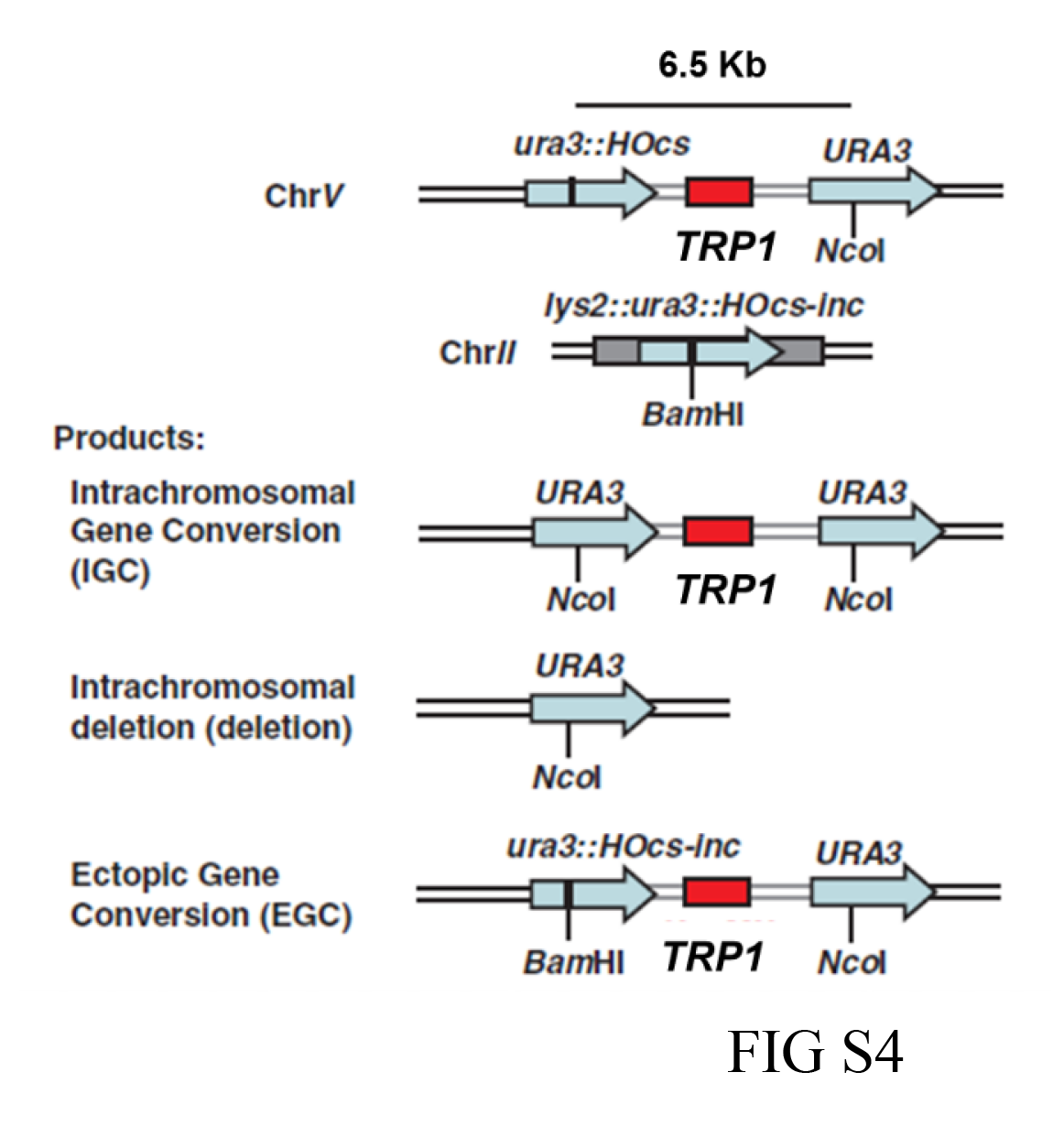

Supplement: FIG S4 [file mbo004183980sf4.tif]
